# Supplementary material for: Splice-Junction-Based Mapping of Alternative Isoforms in the Human Proteome
Source: Cell Rep. Author manuscript; Available in PMC 2020 Jan 15. (PMC6961840; doi:10.1016/j.celrep.2019.11.026)
Supplement: 2 [file NIHMS1546469-supplement-2.zip › DF1/PXD006675/LeftCoronaryArtery/LeftCoronaryArtery_5_SYPL1_ETSLHSPSNTSAPHSQGGIPPPTGL.pdf]

A

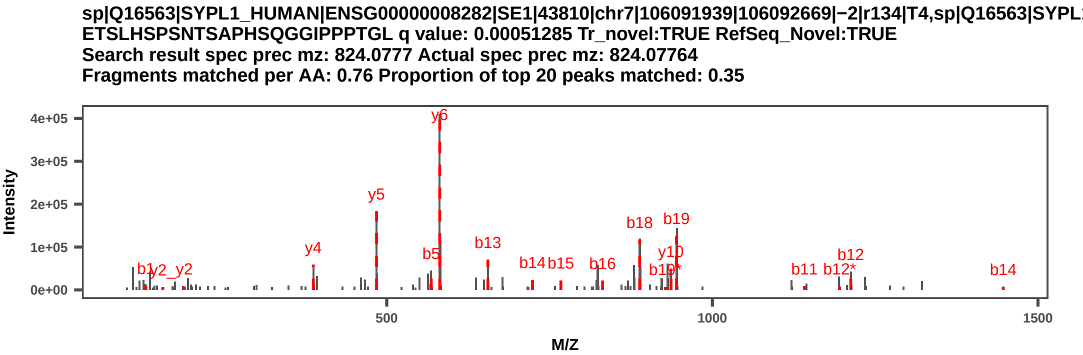
sp|Q16563|SYPL1_HUMAN|ENSG00000008282|SE1|43810|chr7|106091939|106092669|−2|r134|T4,sp|Q16563|SYPL1
ETSLHSPSNTSAPHSQGGIPPPTGL q value: 0.00051285 Tr_novel:TRUE RefSeq_Novel:TRUE
Search result spec prec mz: 824.0777 Actual spec prec mz: 824.07764
Fragments matched per AA: 0.76 Proportion of top 20 peaks matched: 0.35
Intensity
4e+05
3e+05
2e+05
1e+05
0e+00
y6
y5
y4
b5
b13
b18
b19
y10
b19*
b14
b15
b16
b11
b12
b12*
b14
500
1000
1500
M/Z

B

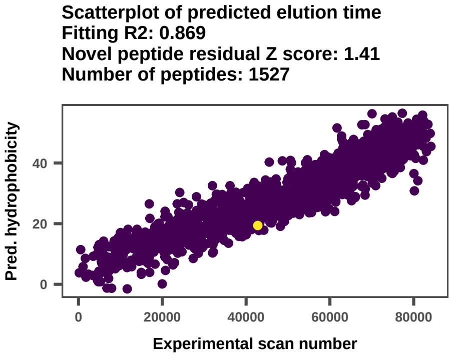
Scatterplot of predicted elution time
Fitting R2: 0.869
Novel peptide residual Z score: 1.41
Number of peptides: 1527
Pred. hydrophobicity
40
20
0
0
20000
40000
60000
80000
Experimental scan number

C

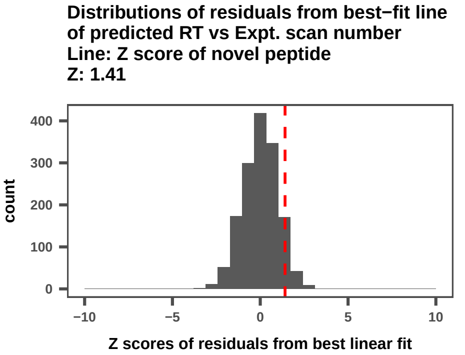
Distributions of residuals from best−fit line
of predicted RT vs Expt. scan number
Line: Z score of novel peptide
Z: 1.41
count
400
300
200
100
0
−10
−5
0
5
10
Z scores of residuals from best linear fit
